# CYP1B1- and CYP1A1-Template systems and their application to metabolism and inhibition

**DOI:** 10.1186/s41021-025-00351-x

**Published:** 2025-12-26

**Authors:** Yasushi Yamazoe, Kaori Ambe, Masahiro Tohkin, Takashi Yamada, Kenichi Masumura

**Affiliations:** 1 Division of Drug Metabolism and Molecular Toxicology, Graduate School of Pharmaceutical Sciences, Tohoku University, 6-3 Aramaki-Aoba, Aoba-ku, Sendai, 980-8578 Japan; 2 Graduate School of Data Science, Nagoya City University, 1 Yamanohata, Mizuho-cho, Mizuho-ku, Nagoya, Aichi, 467-8501 Japan; 3 Graduate School of Pharmaceutical Sciences, Nagoya City University, 3-1 Tanabe-dori, Mizuho-ku, Nagoya, Aich, 467-8603 Japan; 4 Division of Cellular and Molecular Toxicology, Center for Biological Safety and Research, National Institute of Health Sciences, 3-25-26 Tonomachi, Kawasaki-ku, Kawasaki, 210-9501 Japan; 5 Division of Risk Assessment, Center for Biological Safety and Research, National Institute of Health Sciences, 3-25-26 Tonomachi, Kawasaki-ku, Kawasaki, 210-9501 Japan

**Keywords:** CYP1B1-mediated metabolism, Fused-grid Template, Modes of inhibition, Distinction from CYP1A1, Simulation of ligand-interaction on Template

## Abstract

Prediction of cytochrome P450 (CYP)-mediated metabolism is crucial for assessing the safety of chemicals. An in silico system to reproduce CYP1B1-mediated reactions has been developed by the reverse construction of ligand-accessible spaces from ligand assemblies as a fused grid Template* system. There are close similarities between Templates of CYP1B1 and previously established CYP1A1 (Genes & Environ 2023) in the distribution area, Site of oxidation, and the available Width, except for the use of Position 52’ (Pier-sitting) and the lack of use of the bottom in the middle region (Rings F and Ea) on CYP1B1-Template. Experiments using various substrates of both enzymes further suggested the distinct localization of Bay-2 residues on Templates of CYP1A1 and CYP1B1. More than 260 reactions of CYP1A1/1B1 ligands were examined on the present CYP1A1- and CYP1B1-Template systems. From their placements on the Templates and rules for interaction modes, verifications of good and poor substrates, regio/stereo-selectivity, and inhibition became faithfully available for these ligands. To understand the structural basis of the inhibitory interaction, various inhibitors were applied to the Templates and verified modes of the steric interactions. Both the hangings at Position 32 of CYP1A1 ligands and the adherence at Positions 24–52 of CYP1B1 ligands are suggested to retard the dissociation of Bay-2 residues from ligand molecules. Dissociation interference of ligands with Bay-2 residue is thus possible to be a mechanism of ligand-mediated inhibition on CYP1A1 and CYP1B1.

**Supplementary Information:**

The online version contains supplementary material available at 10.1186/s41021-025-00351-x.

## Introduction

Cytochrome P450 (CYP) is a family of enzymes involved in the metabolism of hydrophobic endobiotics and xenobiotics. Assessments of metabolism at individual CYP levels are necessary for efficacy and safety assessments. Despite the development of crystalized CYP-derived 3D models, the predictions of the regio- and stereo-selective metabolisms of small-sized ligands are still far from being established.

CYP1B1, as well as CYP1A1, are expressed polymorphically in the hepatic and extrahepatic tissues in humans. This enzyme mediates the metabolism of endobiotics and xenobiotics, including metabolic activation of promutagens and procarcinogens [1].

Considerable amounts of data on drug metabolism and ligand interactions at individual CYP levels have been obtained from experiments using recombinant human CYP enzyme preparations for more than three decades. With these advantages, in silico systems to reproduce CYP-mediated reactions have been developed by the reverse construction of ligand-accessible spaces from ligand assemblies. Template systems of human CYP enzyme-mediated reactions, constructed from the assembly of the ligands, were refined with the introduction of the allowable width, specific residue, and the residue-initiated movement of ligands in their active sites. These components were in common with Template systems for human CYP1A1 [2, 3], CYP1A2 [2, 4, 5], CYP2B6 [6], CYP2C8 [7], CYP2C9 [8], CYP2C18 [9], CYP2C19 [10], CYP2D6 [11], CYP2E1 [12], CYP2J2 [13], CYP3A4 [14, 15], CYP3A5 [15], and CYP3A7 [15]. These fused grid-based Template systems of human CYPs have been applied to studies of chemical metabolism and safety evaluations due to the advantages of their prediction accuracy and deciphering properties for the possible formation of unstable intermediates and causes of poor metabolisms [2, 4, 16, 17].

In the present study, a Template system of CYP1B1 has been constructed. Applications of various ligands on CYP1B1- and CYP1A1-Templates uncovered the basis of the distinct substrate specificities of CYP1B1 and CYP1A1. In addition, the relationships between ligand placements and the modes of inhibition of CYP1A1 and CYP1B1 were explored using their Templates.

## Materials and methods

Experimental information on the substrate specificities of CYP1A1 and CYP1B1, as well as the metabolism of substrates, was obtained from the literature. The published data on recombinant CYP1A1 and CYP1B1 systems were preferred due to the direct reflections of both enzyme properties.

Chem3D (version 5 for Mac OS, CambridgeSoft, Cambridge, MA), ChemBio3D (version 12 for Windows, CambridgeSoft), and ChemBioDraw (versions 11 and 13 for Mac OS, CambridgeSoft/PerkinElmer) were used to construct two-dimensional (2D) or three-dimensional (3D) structures of ligands and to overlay compounds on Template.

Substrates of CYP1A1 and CYP1B1, except for polyaromatic hydrocarbons (PAHs), take various conformations due to their flexibility. Before Template application, chemicals are altered into their flattened form(s). Conformations of the structures were then modified to fit within Template, considering the bond-energy barrier using MM2 function of Chem3D and specific interactions at distinct regions of the Template. Carbon, oxygen, nitrogen, sulfur, chlorine, and fluorine atoms of 3D ligand structures in figures are indicated with grey, red, blue, yellow, green, and khaki symbols, respectively. The hydrogen atoms of the substrates were not considered for the placement.

Templates consist of hexagonal grids and sticks. The sitting of substrate atoms at each corner of the hexagonal grids (termed Rings) was evaluated as occupancy. Some atoms, placed not precisely at the corner, were accepted if these atoms stayed within the Template area or at specific defined sites. The placement of ligands is expressed in a hyphen-linked form, such as Rings A-B-C, to trace the occupancy of chemical molecules on Template. The branching part is indicated in the bracket. Ligands of CYP1A1 and CYP1B1 were assumed to migrate from Entrance to Site of oxidation without changing the conformation. Thus, ligands enter Template with the same conformations observed at Site of oxidation.

**Fig. 1:**
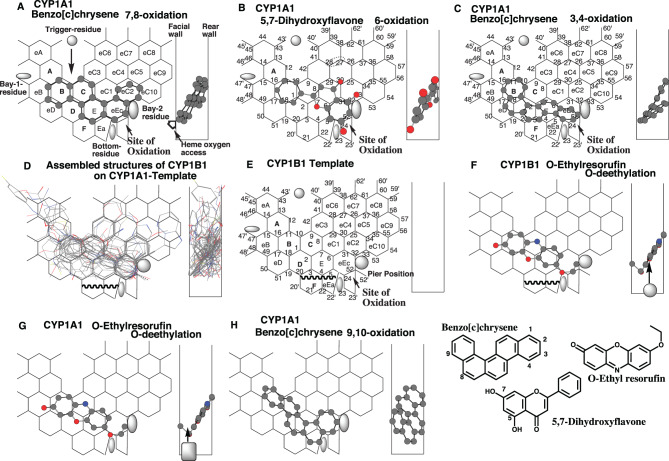
Placements of typical CYP1A1 substrates and ligand assembly of CYP1B1 substrates. Placements of benzo[c]chrysene for the 7,8-oxidation (**A**), 3,4-oxidation (**C**), and 9,10-oxidation (**H**), and of 5,7-dihydroxyflavone 6-oxidation (**B**) are shown as their 3D-structures (cylindrical-shape) on CYP1A1-Template. 90°-rotated view is shown on the right as Width-gauge, which is the allowable depth of the Template. The left and right ends correspond to Facial-wall and Rear-wall, which stand in parallel and are separated by 1.5 Ring-sized distances. Heme-oxygen atom accesses from the facial side on CYP1-Template. Sick-shape structures of CYP1B1 ligands for 20 reactions are arranged with their oxidized parts to sit together in the bottom, and overlaid on CYP1A1-Template (**D**). CYP1B1 Template was extracted from the overlaid construct and assigned Ring names and Position numbers in considering the mutuality on CYP1A1- and CYP1B1-Templates (**E**). Placements of *O-*ethyl resorufin on CYP1B1 and CYP1A1 Templates are shown as their 3D-structures (cylindrical-shape). Round and squire symbols in CYP1B1 and CYP1A1 Width-gauges are Bay-2 residues as described in Fig. 3. 2D-structures are also shown with parts of chemical position numbers

Each part of CYP1A1- and CYP1B1-Templates is identified as Rings and Positions (See Fig. 1 in the section of Results). Facial- and Rear-walls are standing in parallel and separated by 1.5 Ring-sized distances. The allowable depth of Templates is expressed as Width-gauge. Heme-oxygen atom approaches from the facial side to Site of oxidation at Ring eEc of CYP1A1- and CYP1B1-Templates

On CYP1A1- and CYP1B1 Templates, ligands migrate from the top of the template and are finally immobilized by the trigger residue, which appears from the upper point of the rear side. During the process, ligands are necessary to have 1) Simultaneous plural contact with Rear-wall, 2) contact with Facial-wall, 3) contact with Bay-2-residue, 4) interaction with Site of oxidation, and 5) interaction with Trigger-residue for the functional contribution. For Simultaneous plural contact with Rear-wall, placement with a large horizontal distance between contact points is chosen as a candidate. Most ligands fulfill these contacts and interactions as a single molecule (uni-molecule binding). Some ligands lack interaction with either Trigger-residue or Site of oxidation. These ligands are metabolized as bi-molecule binding (pro-metabolized and trigger molecules). For example, phenanthrene is unable to interact simultaneously with Site of oxidation (Ring eEc) and Trigger-site (Ring B) for the 9,10-oxidation. Phenanthrene is expected to undergo the 9,10-oxidation as bi-molecule bindings on CYP1A1/CYP1B1-Templates.

In studies of CYP1A1 and CYP1B1 inhibition, experiments using *O*-ethyl resorufin (ethoxyresorufin) *O*-deethylation (EROD) as a diagnostic marker were employed for ease of comparison.

Chemicals having a lactone moiety are often ionized in neutral pH ranges. These lactones were treated as ionizable groups for the application of substrates in ways similar to other CYP-Template systems [7, 8, 10, 14, 18, 19]. Thus, non-rigid lactone rings are not allowed into contact with Rear-wall and with Trigger-residue of CYP1A1 and CYP1B1 in general.

Several Template-related terms are defined to explain ligand interactions with Template. These terms are listed separately as “Terms used for Template system”

## Results

### General idea for the ligand interactions on CYP1A1-Template and construction of CYP1B1-Template

In the original flat CYP1A1-Template system [3], modes of sittings at Site of oxidation are divided into three sub-types: (i) Orthodox placements (fitting on fused grids) with Hanging to Bay 2 residue (Type 1 Bay 2 interaction) e.g. benzo[c]chrysene 7,8-oxidation at Rings B-C-E-eEc-eC2), (ii) Orthodox placements with sticking on Position 23 (Type 2 Bay 2 interaction, e.g. 5,7-dihydroxyflavone 6-oxidation at Rings B(D)-C-eC1(E)-eEc plus Positions 23 and 33), (iii) Bottom-flattening placements (Type 3 Bay 2 interaction, e. g. benzo[*c*]chrysene 3,4-oxidation at Rings B-D (eD)-E-eEc). The placements of the three substrates were updated in the refined CYP1A1-Template system containing Width-gauge (Fig. 1A-C). These placements fulfilled the essential contact, namely 1) Simultaneous plural contact with Rear-wall, 2) contact with Facial-wall, 3) sitings at Site of oxidation, 4) contact with Bay-2-residue, and 5) contact with Trigger-residue (Fig. 1A).

**Fig. 2:**
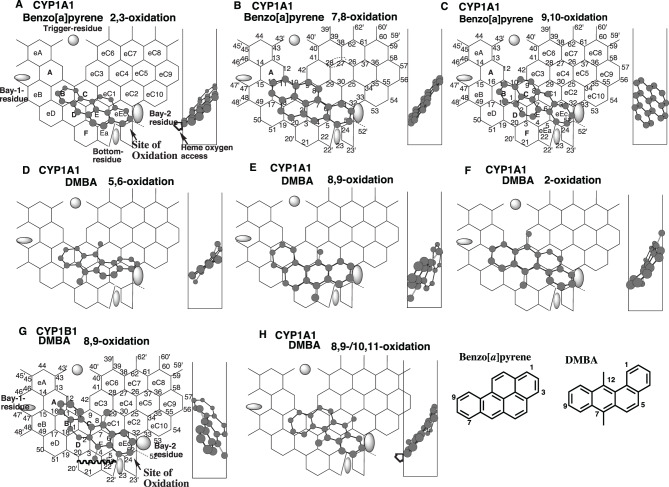
Regioselective oxidations of benzo[a]pyrene and DMBA and their placements. Placements of benzo[a]pyrene for the 2,3- (**A**), 7,8- (**B**) and 9,10- (**C**) oxidations, of DMBA for the 5,6- (**D**), 8,9- (**E**), 2- (**F**) and 9,10/10,11(**H**)-oxidations on CYP1A1-Templates and of DMBA 8,9-oxidation on CYP1B1-Template (**G**) are shown as their 3D-structures (cylindrical-shape). Bay-residues of CYP1B1 (grey oval shape) and CYP1A1(grey square shape) are located near Site of oxidation. 2D-structures are also shown with parts of chemical position numbers

Considering the substrate similarities of CYP1A1 and CYP1B1, the CYP1A1-Template was used for the initial cavity for CYP1B1 substrates.

CYP1B1 substrates were picked up at random (Table 1), and their 3D structures were assembled by considering a certain width between Facial-wall and Rear-wall. Focusing on the positions metabolized at the bottom part resulted in the production of a ligand-accessible space. Assembled structures of CYP1B1ligands were applied on CYP1A1-Template (Fig. 1D).

**Table 1:** CYP1B1 substrates used for Template construction

Ligand name	Reaction	Reference
Aristolochic acid	*O*-demethylation	[20]
Benzo[*c*]chrysene	3,4-oxidation	[21]
	7,8-oxidation	
*R*-Bufuralol	1”-oxidation	[22]
	5,6-oxidation	
Cabozantinib	*N*-oxide formation	[23]
DB289	*O*-demethylation	[24]
Dibenzo[*a,l]*pyrene	11,12-oxidation	[25]
7,12-dimethylbenzo[*a*]anthracene	5,6-oxidation	[26]
Ellipticine	13-oxidation	[27]
Febuxostat	methine oxidation	[28]
Imatinib	*N*-oxide formation	[29]
5-Methylchrysene 1,2-diol	3,4-oxidation	[30]
Linezolid	demethylenation	[31]
Loxapine	*N*-oxide formation	[32]
2-Nitropyrene	nitro reduction	[30]
Nobiletin	*O*-demethylation	[33]
Ponatinib	methyl oxidation	[34]
*trans*-Resveratrol	3’-oxidation	[35]
all-*trans*-Retinal	acid formation	[36]

There are close similarities between CYP1B1 and CYP1A1 in distribution area, Site of oxidation, and Width, except for the use of Position 52’ (Pier-sitting) and the lack of use of the bottom in the middle region (Rings F and Ea). Bay-1 and Bay-2 residues, which were assumed regions of avoidance for ligand sitting, were set from the result of CYP1B1 ligand assembly, similar to those of CYP1A1. A template of CYP1B1 (Fig. 1E) was thus constructed based on the commonalities with those of CYP1A1 [3] and CYP1A2 [18]. The result of ligand assembly suggested that substrates of CYP1B1 did not enter Rings F and eEa (Fig. 1D). Substrates of CYP1B1, but not CYP1A1, sit at Pier Position 52’. Both CYP1A1 and CYP1B1 oxidize substrates mainly at the region connecting Positions 4 and 52 (Site of oxidation) (Fig. 1E). Trigger residue was assumed to appear after the passage of ligands to interact with ligands at Trigger-site (Positions 10–11). Ring name and Position number are mutually used for CYP1A1- and CYP1B1-Template.

**Fig. 3:**
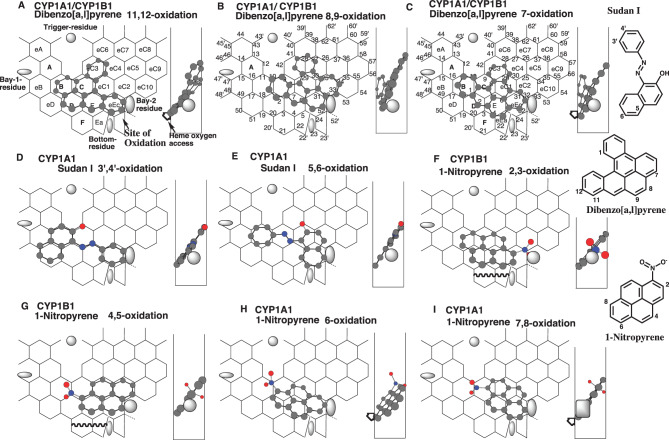
Placements and Bay-2 residue. Placements of dibenzo[*a,l*]pyrene 11,12- (**a**), 8,9- (**b**), 7- (**c**) oxidations, of Sudan I 3’,4’- (**d**) and 5,6- (**e**) oxidations, and of 1-nitropyrene 2,3- (**f**), 4,5- (**g**), 6- (**h**), and 7,8- (**i**) oxidations d are shown as 3D-structures with their 90°-rotated ones in the right sides. The mark of heme oxygen access is added mostly in molecules where the expected site of oxidation is located away from facial-end. Bay-residues of CYP1B1 (grey oval shape) and CYP1A1(grey square shape) are located within width-gauge (right side). CYP1B1 Bay-2 residues (grey oval shape) are shown in width-gauge of CYP1A1, if necessary, to indicate the lack of contact. 2D-structures are also shown with parts of chemical position numbers. Functional and non-functional placements are indicated with dark- and grey-colored structure names, respectively

*O*-Ethyl resorufin *O*-deethylation is often used as a diagnostic marker of both CYP1A1 and CYP1B1 activities. The rates of the *O*-deethylation were, however, more than 10 times higher with CYP1A1 than with CYP1B1 [37]. Placements of *O*-ethyl resorufin were generated at both Ring B (eB)-C(D)-E(eC1)-eEc on CYP1B1- (Fig. 1F) and CYP1A1-Templates (Fig. 1G). The placements fulfilled the essential contact requirements on both CYP1B1- and CYP1A1 Templates. The placements of *O*-ethyl resorufin suggested that distinct rates of CYP1A1- and CYP1B1-mediated *O*-deethylation occurred due to the differences in other areas described above. The contact regions with Bay-2 residues were clarified to be different between CYP1B1 and CYP1A1, as described in detail later. Bay-2 residues of CYP1B1 and CYP1A1 are shown as grey oval and square in Width-gauge (right of Fig. 1F and G).

These properties were examined further with various ligands.

### Placement of PAHs and CYP1A1-selective use of the bottom region

CYP1A1 mediates the 7,8- and 3,4-oxidations of benzo[*c*]chrysene, and slightly the 9,10-oxidation. CYP1B1 also mediates the 7,8- and 3,4-oxidations, but not the 9,10-oxidation [21]. CYP1B1, rather than CYP1A1, shows the higher 3,4- to 7,8-oxidation ratios. The rate of 7,8-oxidation is higher with CYP1A1 in the recombinant systems.

Placements of benzo[*c*]chrysene were generated for the 7,8- 3,4-, and 9,10-oxidations on CYP1A1-Template at Rings B-C-E-eEc-eC2 (Fig. 1A), at Rings B-D (eD)-E-eEc (Fig. 1C), and at Rings B (A)-C-D(E)-Ea-eEc (Fig. 1H), respectively. These placements fulfill the essential contact. The molecule for the 7,8-oxidation had a hanging at Positions 32–33 on CYP1A1-Template (Fig. 1A), while the slide-down to Positions 24–52 at Ring eEc was observed with the molecule for the 3,4-oxidation (Fig. 1C). CYP1A1-selective use of Ring Ea was detected in the placement of benzo[c]chrysene 9,10-oxidation (Fig. 1H), which was consistent with the experimental data.

Benzo[*a*]pyrene undergoes both CYP1A1- and CYP1B1-mediated 2,3-, 7,8- and 9,10-oxidations, although no striking differences were observed in both enzymes’ regioselectivities [38].

Placements of benzo[*a*]pyrene were available for the 2,3-, 7,8- and 9,10-oxidations at Rings B-C (D)-E(eC1)-eEc (Fig. 2A), at Rings B-D-C-E-eEc (Fig. 2B), and at Rings B-C (D)-E(eC1)-eEc (Fig. 2C), respectively. These Type 1 molecules, fulfilling the essential contact, were expected to contact their upper ends with Rear-wall, and also the lower ends with Bay-2 residue at the facial to middle region of Width-gauge of both CYP1A1-Template and CYP1B1-Template lacking Rings F and Ea.

Both CYP1A1 and CYP1B1 mediate mainly 5,6- and 8,9-oxidations of 7,12-dimethylbenzo[*a*]anthracene (DMBA), with a presumed 2-phenol formation and low amounts of the 10,11-oxidation [26].

Placements of DMBA were generated on CYP1A1-Template for the 5,6-, 8,9-, and 2-oxidations at Rings B-C (D)-E(eC1-eC4)-eC1-eC2(eC10) (Fig. 2D), at Rings B-D-E (F)-eEc plus Position 8 (Fig. 2E), and at Rings B-C-eC1-eEc plus Positions 3 and 42 (Fig. 2F), respectively. On the placement of the 5,6-oxidation, the heme-oxygen atom would be able to contact position 5 of the DMBA molecule, which sat at the facial end but above Positions 24–52. Molecules for the 8,9- and 2-oxidations also satisfied the essential contact with CYP1A1-Template.

CYP1B1 mediates the 8,9-oxidation, although the rate was low compared to CYP1A1.

A distinct placement of DMBA was constructed for the CYP1B1-mediated 8,9-oxidation at Rings A-B-C-E (eC1)-eEc plus Positions 4 and 29, and also the facial side of Fjord (Fig. 2G). The 7-methyl part was located at the allowable bottom region of CYP1B1-Template. The higher 7,8-oxidation regioselectivity in recombinant systems of CYP1A1 than of CYP1B1 was possibly related to their distinct placements (Fig.s. 2E and G). A placement of DMBA was generated at Rings B (D)-C(eC3)-E(eC1)-eEc for the minor 10,11-oxidation (Fig. 2H). The DMBA molecule would contribute both 8,9- and 10–11-oxidations and interact with the Bay-2 residue of both CYP1A1 and CYP1B1.

### Placement of PAHs and interaction with Bay-2 residue

CYP1A1 mediates the 11,12-, 8,9- and 7-oxidations of dibenzo[*a,l*]pyrene. CYP1B1 catalyzes the 11,12- and 7-oxidations, but not the 8,9-oxidation [25].

Placements of dibenzo[*a,l*]pyrene were available for the 11,12-, 8,9- and 7-oxidations at Rings B-D (C-eC3)-E-eEc (Fig. 3A), at Rings C (B)-E(eC1-eC3-eC4)-eEc(eC2) (Fig. 3B), and at Rings B-D (C)-E(eC1-eC3-eC4)-eEc (Fig. 3C), respectively.

Ligand molecules must sit stably at the Site of oxidation of CYP1A1- and CYP1B1-Templates. Bay-2 residues, located on the right end of Ring eEc, are expected to support ligand immobilization. The right end part of the ligand molecules on Templates sat between the facial and middle parts of the Width-gauge for the 11,12-oxidation, only at the facial end for the 8,9-oxidation, and at the middle region for the 7-oxidation. These results suggested a possible localization of the CYP1A1 Bay-2 residue in the facial side of the Width-gauge, while the CYP1B1 Bay-2 residue was in the middle region of the Width-gauge. The Bay-2 residue of CYP1B1 was arbitrarily set as a grey oval of 0.5 Ring size in diameter, based on the comparison of experimental data with simulation results of various substrates (Fig. 3A-C). The Bay-2-residue of CYP1B1 was unlikely to contact the molecule of dibenzo[*a,l*]pyrene 8,9-oxidation (Fig. 3 Bright). The molecule for the 7-oxidation barely sat in Ring F and eEa on CYP1B1-Template.

CYP1A1 mediates 3’,4’- and 5,6-oxidations of Sudan I, but CYP1B1 has trace levels of the oxidations [39].

Placements of Sudan I were generated on CYP1A1-Template for the 3’,4’- and 5,6-oxidations at Rings eD-B-C-E-eEc (Fig. 3D), and at Rings B-C-eC1 (eC2)-eEc plus Position 29 (Fig. 3E), respectively. Neither placement use on Rings F/Ea, suggesting a distinct cause for the catalytic differences between CYP1A1 and CYP1B1. Regions expected to contact Bay-2 residue were located only on the facial side of both molecules (Fig. 3D and E, Width-gauge).

The Bay-2 residue of CYP1B1 was thus likely to interact sparingly with the tall Sudan I molecule for both 3’,4’- and 5,6-oxidations, which leaned against the Rear Wall (Fig. 3D and E).

CYP1A1, but not CYP1B1, mediates *N*^2^-oxide formation of ellipticine [27]. The placement at Rings B-C-E(eC1)-eEc, which leaned against the Rear Wall, also suggested the lack of contact of the ellipticine molecule with Bay-2 residue of CYP1B1 (Data not shown).

Both CYP1A1 and CYP1B1 mediate the 3,4- and 5,6-oxidations of benzo[*c*]phenanthrene [40]. The benzo[c]phenanthrene molecules were generated for the 3,4- and for the 5,6-oxidations at Rings B-C(D)-eC3-eC1-eEc (Data not shown), and at Rings C(B)-D-E-eEc(eC1)-eC2 (Data not shown) on CYP1A1- and CYP1B1-Templates, respectively. Both molecules oriented the parts closest to the Bay-2 residue into the facial-to-middle regions of Width-gauge. These results supported the expected localization of CYP1B1’s Bay-2 residue in the middle of Width-gauge, and of CYP1A1’s Bay-2 residue at the facial side of Width-gauge.

1-Nitropyrene undergoes both CYP1A1- and CYP1B1-mediated 2,3-, 4,5-, 6-, and 7,8-oxidations. The rates of 7,8-oxidation were lower with CYP1B1 than with CYP1A1, but the rates of 2,3-oxidation were much higher with CYP1B1 than with CYP1A1 [41].

Placements of 1-nitropyrene were generated for the 2,3-, 4,5-, 6-, and 7,8-oxidations on CYP1A1/CYP1B1-Template at Rings B-D-C (eC1)-E-eEc-eC2 (Fig. 3F), at Rings B (D)-C-E-eC1-eEc-eC2 (Fig. 3G), at Ring B-D-C-E (eC1)-eEc (Fig. 3H), and at Rings B-C-E-eC1-eEc (eC2) (Fig. 3I), respectively.

The Bay-2 residue of CYP1B1 would contact the 1-nitro part at the middle to rear side on the placement of 1-nitropyrene 2,3-oxidation (Fig. 3F Width-gauge). In contrast, the contact difficulty was expected with Bay-2 residue of CYP1A1. These simulation results were consistent with experimental data showing the substantial lack of CYP1A1 activity for the 2,3-oxidation.

The 1-nitro part of 1-nitropyrene contributed to both Trigger interaction and Rear-wall contact in the placement for the 4,5- (Fig. 3G) and 6-oxidations (Fig. 3H). The interactions with Bay-2 residue were expected to occur in the facial and middle regions of Width-gauge, suggesting a functional role for both CYP1A1 and CYP1B1 in both the 4,5- and 6-oxidation.

Consistent with the placement of Sudan I 5,6-oxidation (Fig. 3B), the tall 1-nitropyrene molecule for 7,8-oxidation would have limited contacts with the Bay-2 residue of CYP1B1 (Fig. 3I). The result of the simulation was consistent with the experimental data on the rates of 7,8-oxidation, in which CYP1B1 rather than CYP1A1 showed lower oxidation rates.

Different from 1-nitropyrene, CYP1A1 mediates the 2,3-oxidation of 1-chloropyrene [42]. A placement of 1-chloropyrene was constructed for the 2,3-oxidation at Rings B-D-C(eC1)-E-eEc (Data not shown). The chlorine atom, situated in the middle of the Width-gauge, would interact with the Bay-2 residues of both CYP1A1 and CYP1B1.

The Bay-2 residue of CYP1A1 was arbitrarily set to 0.6 Ring size in diameter, as shown by the grey square on the CYP1A1-Template, based on the comparison of experimental data with simulation results of various substrates (Fig. 3I).

### Placements of substrates showing distinct CYP1A1 and CYP1B1 reactivities

CYP1A1 mediates the 6,7-oxidation and 9’-*N*-demethylation of granisetron [43], but CYP1B1 had only trace levels of the 9’-*N*-demethylase activity [44].

**Fig. 4:**
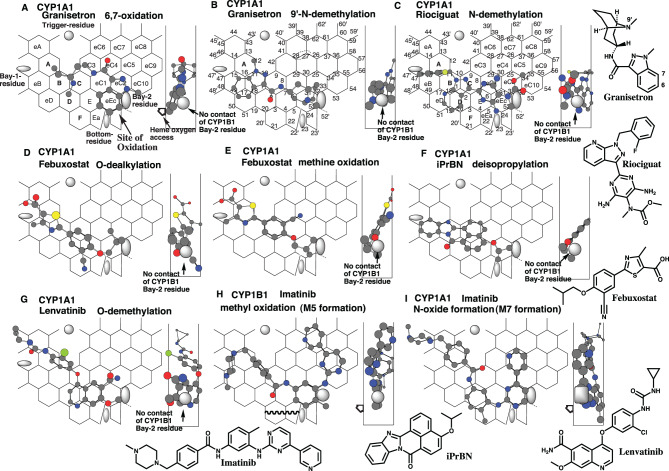
Selective substrates of CYP1A1 and imatinib. Placements of granisetron for the 6,7- (**a**) and 9’- (**b**) oxidations, of riociguat *N*-demethylation (**c**), of febuxostat *O*-dealkylation (**d**) and methine oxidation (**e**), of iPrbn deisopropylation (**f**), of lenvatinib *O*-demethylation (**g**), of imatinib methyl oxidation (**h**) and *N*-oxide formation (**i**) are shown as cylindrical-shapes of 3D-structures on CYP1A1- and/or CYP1B1-Templates- their 90°-rotated placements are shown in width-gauge. Bay-2 residues of CYP1A1 and CYP1B1 are shown as a grey oval and square in width-gauge, respectively. The mark of heme oxygen access is added mostly in molecules where the expected site of oxidation is located away from facial-end. 2D-structures are also shown with parts of chemical position numbers

Placements of granisetron were available on CYP1A1-Template for the 6,7-oxidation n at Rings A-B-C (D)-eC3-eC4-eC2(eC10)-eEc (Fig. 4A), and the 9’-*N*-demethylation at Rings eB (eD)-B(A)-D-C-E-eC1-eEc(eC2) (Fig. 4B). At the Site of oxidation at Ring eEc, both molecules contacted with the Bay-2 residue at the facial end of Width-gauge. The granisetron molecule were not expected to stand in the middle region at Ring eEc for the interaction with Bay-2 residue of CYP1B1.

Riociguat undergoes CYP1A1-mediated, but not CYP1B1-mediated, *N*-demethylation [43].

A placement of riociguat was generated for the *N*-demethylation at Rings eB-A-B (D-eD)-C-eC1(E)-eEc-eC2 on CYP1A1-Template (Fig. 4C). The *N*-methylcarbamate part, stayed at the facial end of Width-gauge, would contact selectively with Bay-2 residue of CYP1A1, but not of CYP1B1.

CYP1A1 mediates the *O*-dealkylation and methine oxidation of febuxostat. CYP1B1 has only the trivial methine oxidase activity.

Placements of febuxostat were available for the *O*-dealkylation and methine oxidation at Rings eA-A-B (eB)-D(F)-C-E-eEc(eC1) (Fig. 4D), and at Rings eA-A (eB)-B-C-eC1(eC4)-eEc (Fig. 4E), respectively. Both molecules achieved simultaneous multiple contacts with the Rear-wall and occupied the Trigger site (Positions 10–11). The facial side orientations at the oxidation site were also necessary for *O*-dealkylation and methine oxidation. These placements enabled contact with the Bay-2 residue of CYP1A1 located on the facial side, but placements of the febuxostat molecule satisfying both facial-side sitting at Site of oxidation and contact with the Bay-2 residue of CYP1B1 were not readily generated after the MM-2 treatment (Data not shown).

CYP1A1, but not CYP1B1, mediates the *O*-de-isopropylation of 4-isopropyl-7*H*-benzo[*de*]benzo [4, 5]imidazole[2,1-*a*]isoquinolin-7-one (iPrBN) [45].

A placement of iPrBN was constructed for the *O*-de-isopropylation at Rings eB-B (eD)-C(eC1)-D-E-eEc (Fig. 4F). Simultaneous plural contact with Rear-wall with positions 1, 2, 11, and 12 resulted in the contacts of the isopropyl group with both Facial-wall and Bay-2 residue of CYP1A1 for the stable sitting. The placement was selective for CYP1A1, and not effective for the interaction with Bay-2 residue of CYP1B1.

Lenvatinib undergoes CYP1A1-mediated, but not CYP1B1-mediated, *O*-demethylation [46].

A placement of lenvatinib was generated for the *O*-demethylation at Rings eA-A-B-C-D (F)-E-eEc (Fig. 4G). Simultaneous plural contact with Rear-wall with the ether bridge part of Lenvatinib forced the facial end localization of the methoxy part for the Bay-2-residue contact. This placement was only feasible for CYP1A1 but not for CYP1B1.

CYP1B1 prefers the methyl oxidation of imatinib’s tolyl part, while CYP1A1 mediates the *N*-oxide formation of the pyrimidine part, the *N*-demethylation, and terminal pyridine-ring oxidation [29].

Placements of imatinib were generated for the methyl oxidation (on CYP1B1-Template), *N*-oxide formation, *N*-demethylation, and terminal pyridine-ring oxidation (on CYP1A1-Template) at Rings eB-A-B-C-D-E-eEc-eC2-eC5 (eC10)-eC7 plus Position 52’ (Fig. 4H), at Rings eA-A-B (C)-D(F)-E-eEc-eC1-eC4 plus a space left of Ring eA (Fig. 4I), Rings eA-A-eB-B-C (D)-eC3-eC4-eC2-eEc (Data not shown), and at Rings eD-eB-B-C-eC3-eC6-(eC7)-eC4-eC2(eC5)-eEc plus a space beneath Ring D (Data not shown), respectively. Pier-sitting to Position 52’ is allowed on CYP1B1, but not on CYP1A1. The placement (Fig. 4H) would explain the causal basis of high CYP1B1’s methyl oxidation. The imatinib molecule (Fig. 4I), sitting at Ring F for the *N*-oxide formation, suggested the CYP1A1 selectivity. The imatinib molecules for the *N*-demethylation and terminal pyridine-ring oxidation (Data not shown) also directed the terminal parts at Ring eEc to their facial sides, which linked to the CYP1A1 selectivity.

### Placements of flavonoids

CYP1B1, but not CYP1A1, mediates the 4’-oxidation of flavone and the subsequent 3’,4’-dihydroxyflavone formation. Instead, CYP1A1 catalyzes the 6-oxidation of flavone [47]. Both reactions proceed slowly and at barely quantifiable rates.

**Fig. 5:**
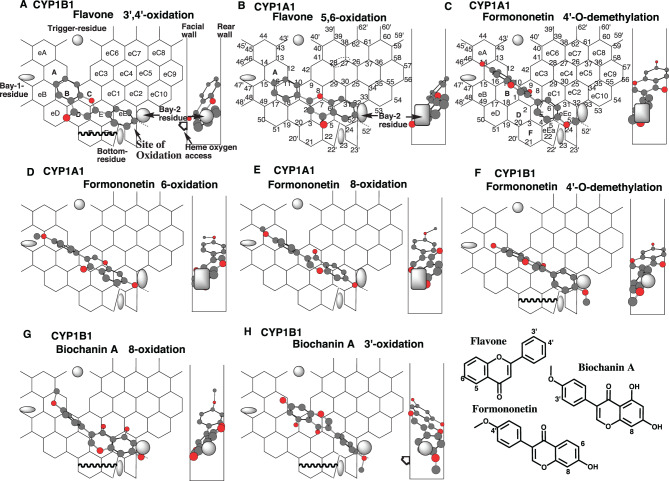
Placements of flavonoids. Placements of flavone 3’,4’-oxidation on CYP1B1-Template (**a**), and 5,6-oxidation on CYP1A1-Template (**b**), of formononetin 4’-*O*-demethylation (**c**), 6- (**d**) and 8- (**e**) oxidations on CYP1A1-Template, 4’-*O*-demethylation on CYP1B1-Template (**f**), of biochanin a 8- (**g**) and 3’- (**h**) oxidations on CYP1B1-Template are shown as cylindrical-shapes of 3D-structures on Template. Their 90°-rotated placements are shown in width-gauge. Bay-2 residues of CYP1A1 and CYP1B1 are shown as a grey oval and square in width-gauge, respectively. 2D-structures are also shown with parts of chemical position numbers. The mark of heme oxygen access is added mostly in molecules where the expected site of oxidation is located away from facial-end

A placement of flavone was available for the 4’-oxidation of flavone at Ring B-C(eC3)-eEc. The phenyl part would be fastened at Positions 24–52 on CYP1B1-Template (Fig. 5A), but not on CYP1A1-Template (Data not shown). Futile-sitting of the phenyl group is possible to occur on the flavone molecule on the CYP1A1-Template. Similar phenomena are expected on the rotatable and non-substituted phenyl group of CYP3A4 ligands [48].

A distinct placement of flavone was generated for the 5,6-oxidation on CYP1A1-Template at Ring B-C-E (eEa)-eEc (Fig. 5B). The firm sitting of the phenyl-g-pyrone would result in the fixation of the 2-phenyl part at Trigger-site. 4-Keto part of the molecule stayed within the bottom of Ring E. The placement would thus serve for the 5,6-oxidation on the Template.

CYP1A1 mediates the 4’-*O*-demethylation, 6- and 8-oxidations, and also traces of 3’-oxidations of formononetin (7-hydroxy-3-(4-methoxyphenyl)-4*H*-chromen-4-one), but CYP1B1 mediates only the *O*-demethylation of this isoflavone [49].

Placements of formononetin were generated on CYP1A1-Template for the 4’-*O*-demethylation, 6- and 8-oxidations at Rings A-B-C-E-eEc (Fig. 5C), at Rings A-B-C (D)-E(eC1)-eEc (Fig. 5D), at Rings A (eA)-B-C-E(eC1)-eEc (Fig. 5E), respectively.

A placement of formononetin was available on CYP1B1-Template for the 4-*O*-demethylation at Ring A-B-C-B (eC1)-eEc plus Position 52’ (Fig. 5F). The formononetin molecule satisfied Simultaneous plural contact, Facial-wall contact, and Bay-2 residue contact with the methoxy part. Placements for the 6- and 8-oxidations of formononetin were also constructed on CYP1B1-Template at Rings A-B-C-E(eC1)-eEc plus Position 52’ (Data not shown) and at Ring A-B-C-E(eC1)-eEc plus Position 52’ (Data not shown). The rotatable methoxyphenyl parts located at Trigger-site (Ring B), however, hampered both placements on CYP1B1-Template.

Biochanin A (5-hydroxy-formononetin) undergoes CYP1B1-mediated 8- and 3’-oxidations.

Placements of biochanin A were generated for the 8- and 3’-oxidations at Rings A (eA)-B-C-E(eC1)-eEc(eC2) plus Position 52’ (Fig. 5G), and at Rings eB-B-C (D)-E-eEc(eC1)-eC2 (Fig. 5H), respectively. The 5-hydroxy groups of biochanin A molecules contributed to their Rear-wall contacts and supported their stable binding on CYP1B1-Template (See Table 2).

**Table 2:** Terms used for Template system

**2D and 3D:** two-dimensional and three-dimensional
**Bay 1 and Bay 2:** Residues located lower left and right of CYP1A1- and CYP1B1-Templates
**Bi-molecule and uni-molecule binding:** Interactions on Template with Trigger- and Pro-metabolized molecules combination, and with a single molecule
**Entrance:** Routes for the entry of ligands
**Essential contact:** Interactions with CYP1 Templates for ligand immobilization, which consist of 1) Simultaneous plural contact with Rear-wall, 2) contact with Facial-wall, 3) sitings at Site of oxidation, 4) contact with Bay-2-residue, and 5) contact with Trigger-residue
**Fjord:** A central space where no substrate stays on CYP1 Template
**Futile-sitting:** A phenomenon of lack of oxidation associated with rotatable and non-substituted phenyl group of ligands
**Hanging:** Sitting at Position 32 of CYP1A1 ligands
**Pier-sitting:** Sticking of ligand part at Position 52’
**Pro-metabolized molecule:** Substrates to be oxidized or reduced are termed “pro-metabolized molecule” in the simulation experiment
**Simultaneous plural-point contact:** Ligands initiate interaction with Template from their simultaneous contact at the plural parts to Rear-wall.
**Site of oxidation:** A confined space of enzymatic catalysis to interact with heme
**Trigger molecule:** A molecule, which is not oxidized, acts to trigger the catalysis. Trigger molecules need to have an overlap with pro-metabolized molecules on Template
**Trigger-site:** A site to which **Trigger residue** moves to hold ligands to initiate catalyses. For example, Positions 10–11 of CYP1A1 and CYP1B1
**Type-1, Type-2 and Type-3 placements on CYP1A1:** Ligands hang on Bay 2 residue through sitting at Position 33 of Ring eC2 on Type-1 placement. Ligands having a sticking part on Ring eEa on Type-2 placement. Ligands take bottom-flattened sittings contacting simultaneously with the side part to Bay 2 residue and with the flat part to Bottom-residue on Type-3 placement
**Uni-molecule and bi-molecule binding:** Placements on Template with single molecule and with Trigger and Pro-metabolized molecules, respectively
**Width-gauge:** A guide tool to verify the allowable width for ligand accommodation around Template, which was determined empirically

### Placement of steroids

Estrone undergoes CYP1A1-mediated 2-, 4-, and slightly 16a-oxidations. CYP1B1 also mediates 2- and 4-oxidations [50, 51].

**Fig. 6:**
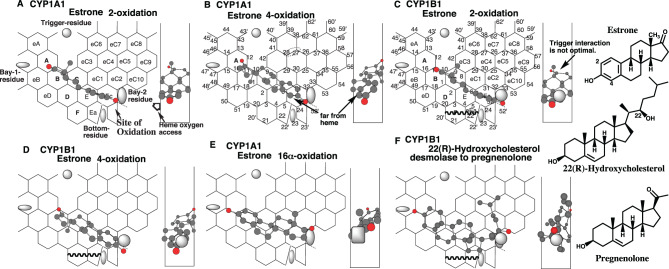
Placements of steroids. Placements of estrone 2- (**a**) and 4- (B)-oxidations on CYP1A1-Template, 2- (**c**) and 4- (**d**) oxidations on CYP1B1-Template, and 16a-oxidation on CYP1A1-Template (**e**), and of 22(R)-hydroxycholesterol sidechain oxidation (**f**) are shown as cylindrical shapes of 3D-structures on Template. Bay-2 residues of CYP1A1 and CYP1B1 are shown as a grey oval and square in width-gauge, respectively. 2D-structures are also shown with parts of chemical position numbers

Placements of estrone were available for the 2- and 4-oxidations on CYP1A1-Template at Rings B (A)-C-E-eEc (Fig. 6A), and at Rings A-B-C-eC1 (E)-eEc (Fig. 6B), respectively. Sitting of 18-methyl at facial side of Position 10 for the 2-oxidation was unlikely to disturb the trigger-residue interaction. Placements on CYP1B1-Template were also generated for the 2- and 4-oxidation at Rings B-C (eC3)-E-eEc plus Position 52’ (Fig. 6C), and at Rings B (A)-C-eC1(E)-eEc plus Position 52’ (Fig. 6D), respectively. A placement of estrone for the 16a-oxidation was also constructed on CYP1A1-Template at Rings eB-B-D (C)-eC1(E) plus a space near Position 32 (Fig. 6E).

CYP1B1 mediates the 4-oxidation of estrone at higher rates than does the 2-oxidation, although both the 2 and 4 positions of estrone A-ring are situated close enough for heme-oxygen access (Fig. 6C and D). Both molecules, however, differed in their sittings at the Trigger Site (Positions 10–11). The 17-keto part of the ring D was situated at Rear-wall for the 4-oxidation (Fig. 6D), but on the facial side for the 2-oxidation (Fig. 6C). These differences are likely to affect the efficiency of trigger-interaction. No Pier-sitting of the 3-hydroxy part existed at Site of oxidation (Ring eEc) on the CYP1A1 placement. Trigger interaction would occur without difficulty on CYP1A1-mediated estrone oxidation (Fig. 6A and B). The 4-position of the estrone ring A was located away from the facial end of CYP1A1 (Fig. 6B). The steric difference would affect the reducing rate of CYP1A1-mediated 4-oxidation of estrone.

22(*R*)-Hydroxycholesterol undergoes CYP1B1-mediated cleavage of the side chain to pregnenolone [52].

A placement of 22(*R*)-hydroxycholesterol was generated for the side-chain cleavage at Rings A-B-D (C)-E(eC1)-eEc-eC2(eC10)-eC4(eC5) on CYP1B1-Template (Fig. 6F). The molecule would sit stably with the contact of CYP1B1’s Bay-2-residue at the middle of Width-gauge.

Placements of CYP1 and CYP1B1 substrates described above suggest three distinct causal bases for the dissimilar catalyses of CYP1A1 and CYP1B1.

1. Selective use of Ring F/Ea region on CYP1A1-Template,
2. CYP1A1 Bay-2 residue (shown as a square shape 0.6 R in diameter) is located between the facial to middle region of Width-gauge. CYP1B1 Bay-2 residue (shown as an oval shape 0.5 R in diameter) is located in the middle region of Width-gauge.
3. CYP1A1 ligands do not exceed the line 24–52 of Ring eEc, while CYP1B1 ligands exceed the line and/or Pier-sitting around Position 52’.

### Placements of intense inhibitors

7,8-Benzoflavone (a-naphthoflavone) is an inhibitor of CYP1 enzymes use for diagnostic purposes. This chemical inhibited CYP1A1- and CYP1B1-mediated EROD with IC_50_ of 60 nM and 9 nM, respectively [53].

**Fig. 7:**
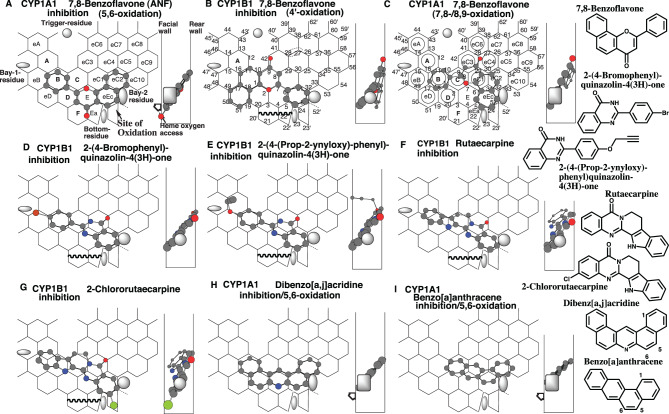
Placements of inhibitors. Placements of 7,8-benzoflavone for the inhibition (5,6-oxidation) (**a**), inhibition (4’-oxidation) (**b**), 7,8/8,9-oxidation (**c**), of 2-(4-bromophenyl)-quinazolin-4(3*H*)-one for the inhibition (**d**), of 2-(4-(prop-2-ynyloxy)-phenyl)-quinazolin-4(3*H*)-one for the inhibition (**e**), of rutaecarpine for the inhibition (**f**), of 2-chlororutaecarpine for the inhibition (**g**), of dibenzo[*a,j*]acridine for the inhibition/5,6-oxidation (**h**), and benzo[*a*]anthracene for the inhibition/5,6-oxidation (**i**) are shown as cylindrical-shapes of 3D structures with their 90°-rotated placements in each right-side. Bay-2 residues of CYP1A1 and CYP1B1 are shown as a grey oval and square in width-gauge, respectively. The mark of heme oxygen access is added mostly in molecules where the expected site of oxidation is located away from facial-end. 2D-structures are also shown with parts of chemical position numbers

Placements of 7,8-benzoflavone were generated for the inhibition of CYP1A1 and CYP1B1 at Rings B-D-E (F)-eEc-eC2 (Fig. 7A), and at Rings eD-B-C (eC3)-eC1-eEc (Fig. 7B), respectively.

Another placement was constructed at Rings B-C-eC1 (eC3)-eC2-eEc (Fig. 7C). Bay-2 residue of CYP1A1, but not of CYP1B1, would be able to contact this molecule.

These 7,8-benzoflavone molecules would be held tightly as suggested by the essential contact (Simultaneous plural contact with Rear-wall, Facial-wall contact, sitings at Site of oxidation, and at Trigger-site, and also interaction with Bay-2-residues) on Templates. The placement of the first, second, and third molecules corresponded to the 5,6-, 4’- and 7,8/8,9-oxidations of this ligand. Although not the data of human CYP1A1, rat CYP1A1 (P450c) mediates the 7,8-oxidation at higher rates than did the 5,6-oxidation [54]. These data might indicate a rather concrete interaction of CYP1A1 for the 5,6-oxidation of 7,8-benzoflavone, namely the tight interaction of Bay-2 residue with the first molecule (Fig. 7A) by way of hanging at Position 32. CYP1A1 selectivity of the first molecule was also suggested from the sitting at Ring F. From the localization of Bay-2 residues, the second molecule, which contacts the Position 24–52 line on the facial side (Fig. 7B), was expected to serve as a CYP1B1 inhibitor. No 4’-hydroxy metabolite is detected as a microsomal 7,8-benzoflavone metabolite [54]. CYP1A1 might be unable to mediate the 4’-oxidation of 7,8-benzoflavone, similar to the case with flavone 4’-oxidation [47]. The third molecule might also contribute to CYP1B1 inhibition after pushing out of the 8-position outside of Ring eEc.

Placements hanging at Position 32 of Ring eEc were often detected on CYP1A1 ligands, while placements contacting the facial side of Positions 24–25 at Ring eEc and/or Pier-sitting to Position 52’ were observed on CYP1B1 ligands. Various CYP1A1 and CYP1B1 inhibitors were thus applied to define the structural interactions on Templates.

## Striking inhibition of CYP1B1

### Quinazolinones

2-Phenylqunazolin-4-ones inhibit CYP1A1- and CYP1B1-mediated EROD [55, 56]. Several quinazolinones showed much stronger inhibitions for CYP1B1 (IC_50_ of 2–21 nM) than for CYP1A1 (IC_50_ of > 1,000 nM).

Placements of the 4-bromo (IC_50_ of 3 nM) and 4-propynyloxy derivatives (IC_50_ of 2 nM) were generated on CYP1B1-Template at Rings eB-B-C-E (eC1)-eEc (Fig. 7D), and at Rings A-eB-B-C-E (eC1)-eEc (Fig. 7E), respectively. Both molecules leaning against the rear wall were not hung on Bay-2 residues, but stayed tightly with the facial side of Positions 24–52.

Several rutaecarpine and its derivatives inhibit CYP1B1 [57]. The quinazolin-4-one structure was included in their molecules, suggesting the participation in their rear-wall contact.

Placements of rutaecarpine (IC_50_ of 55 nM) and 2-chlororutaecarpine (IC_50_ of 37 nM) were constructed on CYP1B1-Template at Rings A (eB)-B-C-E(eC1)-eEc (Fig. 7F), and at Rings eD (eB)-D(B)-C-eC1(E)-eEc(eC2) plus Position 52’ (Fig. 7G), respectively. Flipping of the quinazoline and indole rings generated a distinct placement of rutaecarpine, at Rings eB(eD)-B(D)-C-E-eEc, but not for 2-chlororutaecarpine on CYP1B1-Template due to the protrusion of the chlorine atom to a space outside of Ring eD (Data not shown).

### Stilbenes and biphenyl ureas

Methoxy stibenes showed more inhibition of CYP1B1 than of CYP1A1 [58, 59]. IC_50_ values for EROD of 2,4,2’,6’-tetramethoxy-*trans*-stilbene and 3,4,2’-trimethoxy-*trans*-stilbene were 1.77 nM and 3.4 nM, respectively.

Placements of 2,4,2’,6’-tetramethoxy- and 3,4,2’-trimethoxy-*trans*-stilbenes were generated at Rings B (eD)-C(eC3)-E-eEc(eC2) plus a space around Position 52’ (Supplement Fig. 1A), and at Rings A-B (eB)-C-E-eEc(eC2) (Supplement Fig. 1B), respectively. Methoxyphenyl parts of these stilbenes contacted Positions 24–52 line and took a thin plate shape at Ring eEc.

CYP1B1 inhibitors, pifithrin (IC_50_ of 20.63 nM) and biphenyl urea such as 1-(3-chlorophenyl)-3-phenylurea (IC_50_ of 5 nM) [60], also took slope-shape placements leaning against Rear-wall at Rings A (eA)-B-C-eC1(eC3)-eEc (Supplement Fig. 1A), and at Rings A-B-C-E-eEc plus around Position 52’ (Supplement Fig. 1B).

### PAHs

Dibenzo[a,h]anthracene inhibits CYP1B1-mediated EROD with an IC_50_ of 5.2 nM, and CYP1A1-mediated EROD with an IC_50_ of > 1,000 nM [53].

A placement of dibenzo[*a,h*]anthracene was available for the inhibition at Rings A-B-C-eC1-eEc (Supplement Fig. 1C) on CYP1B1-Template. A distinct placement hanging at Position 32 was constructed at Rings B (eB)-C-E-eC1(eEc)-eC2 (Supplement Fig. 1D), but lacked the occupancy at Site of oxidation.

3-Methylcholanthrene inhibited CYP1B1-mediated EROD intensely (IC_50_ of 14 nM), but scarcely the CYP1A1-mediated activity (IC_50_ of > 1,000 nM) [53].

Placements of 3-methylcholanthrene were available for the inhibitions at Rings B-C (eC1)-E-eEc(eC2) plus Position 52’ on CYP1B1-Templates (Supplement Fig. 1E), and at Rings B-CeC1-eC2-eEc plus Position 33 (Supplement Fig. 1F) on CYP1A1-Template. Both the placements satisfied Bay-2 residue contacts. The former molecule took a placement leaning against Rear-wall, which was consistent with placements of CYP1B1 inhibitors described above. The latter molecule, with the hanging Bay-2 residue, was expected to relate to the 4.5-oxidation, but not to inhibit intensely due to the lack of tight contact at Site of oxidation. A distinct placement was also generated at B-C(D)-E-eC1-eEc-eC2 plus Position 53 (Data not shown), but was rather related to the 1,2-oxidation.

Benzo[*b*]fluoranthene inhibits CYP1B1-mediated EROD with an IC_50_ of 4.9 nM, and CYP1A1-mediated EROD with an IC_50_ of 250 nM [53].

Placements of benzo[*b*]fluoranthene were generated on CYP1B1-Template at Rings B (D)-C(eC3)-eC1(E)-eEc (Supplement Fig. 1G), and on CYP1A1-Template at Rings C (B)-eC1(E)-eEc-eC2(eC10) (Supplement Fig. 1H).

The former molecule took a placement leaning against Rear-wall and tight contact with the Position 24–52 line, which was consistent with properties of CYP1B1 inhibitors. The latter molecule had a Bay-2 residue interaction with the 4-position (Supplement Fig. 1H). The second and third molecules were, thus, possible to inhibit CYP1A1.

### CYP1A1 inhibition and placement

Dibenz[*a,j*]acridine inhibits CYP1A1-mediated EROD with an IC_50_ of 56 nM, although this chemical inhibited the CYP1B1-mediated activity with an IC_50_ of 15 nM [53]. A placement of dibenz[*a,j*]acridine was available at Rings B-D-E-eEc-eC2 on both CYP1A1- and CYP1B1-Tamplates (Fig. 7H). The molecule had a hanging group at Position 32 and contacted the Positions 24–52 line, which made a placement feasible for interaction with both CYP enzymes.

Benzo[*a*]anthracene inhibits CYP1A1-mediated EROD with an IC_50_ of 170 nM, and CYP1B1-mediated EROD with an IC_50_ of 9.1 nM [53].

Placements of benzo[*a*]anthracene were constructed at Rings C (B)-eC1(E)-eEc-eC2(eC10) with hanging at Position 32 on CYP1A1-Template (Fig. 7I), and at rings B-C-eC1-eEc with contacting Positions 24–52 line on CYP1B1-Template (Data not shown). The former and latter molecules corresponded to the placements for the 5,6- and 2,3-oxidations, respectively. The former’s occupancy at Ring eEc might, however, suggest the insufficiency for the inhibitory action, similar to the case with 3-methylcholanthrene (Supplement Fig. 1F). As a cause of the clear differences between human and rat CYP1A1-mediated oxidation of dioxin and its chloro-derivatives [61], a dibenzo-*p*-dioxin molecule was suggested to take a sitting at Rings F-Ea (E)-eEc(eC1)-eC2 (Supplement Fig. 1I) on human, but not rat, CYP1A1-Template [3]. An oxygen atom of the dibenzo-*p*-dioxin, located at Position 24 of Ring eEc, interacted with heme for the inhibition of human CYP1A1.

Benzo[*a*]anthracene might take a human CYP1A1-selective dioxin-type placement [3] for the inhibition at Rings F (D)-E(Ea)-eEc-eC2(eC1) (Fig. 7I). Both the uni- or bi-molecule binding was possible to be a mechanism of the inhibition.

Dibenz[*a,c*]anthracene inhibits CYP1A1-mediated EROD with an IC_50_ of 130 nM, and the CYP1B1-mediated activity with an IC_50_ of 8.8 nM [53].

Placements of dibenz[*a,c*]anthracene were generated at Rings B-C (D)-E(F)-eC1(eEc)-eC2 (Supplement Fig. 2A) and at Rings eD-B (D)-C(eC3-eC4-eC7-eC6)-eC1-eEc(eC2) (Data not shown) on CYP1A1-Template, at Rings B-C-eC1(eC2-eC4)-eEc on CYP1B1-Template (Supplement Fig. 2B), and at Rings B (D)-C(eC3)-E(eC1)-eEc on CYP1A1-Template (Supplement Fig. 2C). The second, third, and forth molecules corresponded to placements of the 2,3-, 2,3-, and 10,11-oxidations. Judging from the hanging at Position 32 and pushing at Positions 24–52, the first and third placements would contribute to the inhibition of CYP1A1 and CYP1B1, respectively.

Benzo[*a*]pyrene inhibits CYP1A1-mediated EROD with an IC_50_ of 350 nM, and the CYP1B1-mediated activity with an IC_50_ of 31 nM [53].

Placements of benzo[*a*]pyrene were constructed at Rings B-C (E)-eC1-eEc(eC2) on CYP1A1-Template (Supplement Fig. 2D), and at Rings B-D (C)-E-eEc on CYP1B1-Template (See Fig. 2B) in considering the inhibitory interactions. The former and latter molecules corresponded to placements of the 4,5-oxidation and of the 7,8-oxidation, respectively.

Galangin (3,5,7-trihydroxyflavone) inhibits CYP1A1-mediated EROD with an IC_50_ of 65 nM, and the CYP1B1-mediated activity with an IC_50_ of 8 nM [53].

Placements of galangin were generated at Rings B-C-E-eC1 (eC3)-eEc plus Positions 23, 30, and 33 on both CYP1A1- and CYP1B1-Templates (Supplement Fig. 2E). Another placement was constructed at Rings A-B-C (D)-E(F)-eEc plus Positions 23 and 33 on CYP1A1-Template (Supplement Fig. 2F). Both molecules corresponded to the 6-oxidation placements. The latter had no sitting at the facial side of Ring eEc, and thus would not be effective for inhibition.

Acacetin inhibits both CYP1A1- and CYP1B1-mediated EROD, with IC_50_ of 88 nM and 12 nM, respectively [62].

A placement of acacetin was available at Rings eB-B-C-eC1 (E)-eEc plus Positions 23 and 33 (Supplement Fig. 2G). Both Bay-2 residues of CYP1A1 and CYP1B1 interacted with the flavonoid 6-position of this molecule.

Diosmetin (4’-*O*-methyl-luteolin) inhibits CYP1A1-mediated EROD with an IC_50_ of 140 nM, and CYP1B1-mediated EROD with an IC_50_ of 29 nM. Luteolin inhibits CYP1A1-mediated EROD with an IC_50_ of 1,249 nM, and the CYP1B1-mediated EROD with an IC_50_ of 79 nM [62].

A placement of diosmetin was available at Rings eB-B (eD)-C-eC1(E)-eEc plus Positions 23 and 33 (Supplement Fig. 2H). The methoxyphenol part at Ring B interacted with the Rear wall, and the 5-hydroxy part interacted with the Bay-2 residue on the CYP1A1 Template. A placement of luteolin was generated at Rings eB-B (eD)-C-eC1(E)-eEc plus Positions 23 and 33 (Supplement Fig. 2I). The 4’-hydroxy, but not the catechol, part of luteolin worked for Simultaneous plural contact with Rear-wall. The differences in the Rear-Wall contact of luteolin might relate to the diminished affinity of CYP1A1. These simulation results suggested the influence of the Trigger-residue interaction on the inhibitory action.

## Discussion

The CYP1B1-template system has been established through the assembly of ligands (Fig. 1D) and comparison of ligand interactions with those of CYP1A1 (Fig. 1E).

Currently, 267 reactions of 214 distinct chemicals, reported as CYP1B1 ligands, were examined in the CYP1B1-Template system. These include 137 good (oxidations/reductions), 42 poor catalyses, and 105 inhibitions. Their molecular masses range between 131 (3-methylindole [63]) and 532 (ponatinib [34]). Placements of ligands on CYP1B1-Template were available for all the reactions of good catalysis and inhibition. Verifications of good and poor substrates, and of regioselectivity and stereoselectivity, were obtained steadily by the applications of 3D ligand structures on CYP1B1-Template, with more than 99% accuracy.

CYP1B1 and CYP1A1 share a broad range of their substrate specificity, but clear distinctions are also known in their regioselectivity. Applications of ligands on both CYP1A1- and CYP1B1-Templates suggested three causes of the distinct catalysis. 1. CYP1A1, but not CYP1B1, molecules sat on Ring F or Ea (benzo[*c*]chrysene 9,10-oxidation (Fig. 1E), febuxostat *O*-dealkylation (Fig. 4D), and imatinib *N*-oxide formation (Fig. 4I)). 2. CYP1A1 contacted ligands with the Bay-2 residue located at the facial side of Width-gauge, while CYP1B1 contacted ligands with the Bay-2 residue located in the middle (dibenzo[*a,l*]pyrene 8,9-oxidation (Fig. 3 B), granisetron 6,7-oxidation (Fig. 4A) and 9’-*N*-demethylation (Fig. 4B)). 3. CYP1A1 ligands do not exceed the line 24–52 of Ring eEc, CYP1B1 ligands exceed the line and/or do Pier-sitting around Position 52’, if necessary (Estrone 2/4-oxidations (Fig.s. 6A-D), formononetin oxidations (Fig.s. 5C and F)). These distinct uses of Template regions and of Bay-2 residues would thus result in the different catalyses of CYP1A1 and CYP1B1.

Considerable amounts of ligand data have been accumulated on CYP1A1 and CYP1B1.

No clear explanation has yet been provided on the mechanistic distinction between the substrates and inhibitors. To understand the structural requirements of the inhibitory interaction on both CYP1 enzymes, various intense inhibitors were applied to the Templates and verified modes of the steric interactions.

A placement hanging at Position 32 of CYP1A1-Template was suggested for the inhibition of 7,8-benzoflavone (Fig. 7A). The placement also fulfilled essential interactions for substrates, namely Simultaneous plural contact with Rear-wall, contact with Facial-wall, interactions with Trigger-residue, Bay-2-residue, and also with heme at the Site of oxidation.

Chemicals inhibiting CYP1A1 with an IC_50_ value of less than 500 nM (EROD), except the heme ligand like ellipticine [64], were able to take placements hanging at Position 32 and fulfilling the essential contact on CYP1A1-Template. These included dibenz[*a,j*]acridine (Fig. 7H), benzo[*a*]anthracene (Fig. 7I), benzo[*b*]fluoranthene (Supplement Fig. 1H), benzo[k]fluoranthene (Rings B-C-E(eC1)-eEc-eC2, data not shown), benzo[j]fluoranthene (Rings B-C-eC1(eC3)-eEc(eC2), data not shown), dibenz[*a,c*]anthracene (Supplement Fig. 2A), benzo[*a*]pyrene (Supplement Fig. 2D), galangin (Supplement Fig. 2E), acacetin (Supplement Fig. 2G), and diosmetin (Supplement Fig. 2H), 5-methylchrysene (Rings B-D-C(E)-eC1-eEc(eC2), data not shown), 1-amino-4-chloro-2-methylanthracene-9,10-dione (Rings B-C-eC1(E)-eEc(eC2), data not shown), cannabidiol (Rings A(eA)-B-C(eC1)-E-eEc plus Position 33, data not shown), 2,4,3’,5’-tetramethoxy-*trans*-stilbene (Rings A-B(eD)-C(D)-E-eEc(eC2)-eC1-eC4, data not shown) and 1-ethnylpyrene (Rings B-D(C)-E-eC1-eEc(eC2), data, not shown). All these placements fulfilled essential interactions for substrates and interacted tightly with CYP1A1’s Bay-2 residue. These results suggest that the immobilization of Bay-2 residue, through Position 32 hangings, is associated with intense CYP1A1 inhibition of ligands.

Various ligands like PAHs, quinazolones, stilbenes, and flavonoids inhibit CYP1B1 with low inhibitory constants. These CYP1B1 inhibitors sit in a mutual shape on Ring eEc. CYP1B1 inhibitors such as 2-(4-bromophenyl)-quinazolin-4(3*H*)-one (Fig. 7D), rutaecarpine (Fig. 7F), and 2,4,2’,6’-tetramethoxy-*trans*-stilbene (Supplement Fig. 1A) contact line 24–52 of Ring eEc and Bay-2 residue, and lean against Rear-wall. This type of placement was also observed on PAHs like dibenzo[*a,h*]anthracene (Supplement Fig. 1C), 3-methylcholanthrene (Supplement Fig. 1E), and galangin (Supplement Fig. 2E). This mode of interaction suggests the adherence of the plate-like shape of CYP1B1 ligands with Bay-2 residue at Ring eEc.

In this study, various inhibitors were applied to the Templates and examined modes of the steric interactions. Two common features became apparent on the Templates. These interactions, hangings at Position 32 of CYP1A1 ligands and adherence at Positions 24–52 of CYP1B1 ligands, suggested the retarded dissociation of the Bay-2 residue from ligand molecules. Interference with Bay-2 residue dissociation is thus possibly a mechanism of ligand-mediated inhibition on CYP1A1 and CYP1B1. Of course, further study is necessary to prove the mechanism.

This research was partially supported by the research grant of the Ministry of Health, Labour, and Welfare of the Japanese Government (K. Ambe, Grant Number 24KD2004).

## Electronic supplementary material

Below is the link to the electronic supplementary material.

Supplementary Material 1

Supplementary Material 2

## Data Availability

No datasets were generated or analysed during the current study.
