# Supplementary material for: CYP1B1- and CYP1A1-Template systems and their application to metabolism and inhibition
Source: Genes Environ. 2025 Dec 26;48:1. doi: 10.1186/s41021-025-00351-x (PMC12781794; doi:10.1186/s41021-025-00351-x)
Supplement: Supplementary file 1 — Supplementary Material 1 [file 41021_2025_351_MOESM1_ESM.pdf]

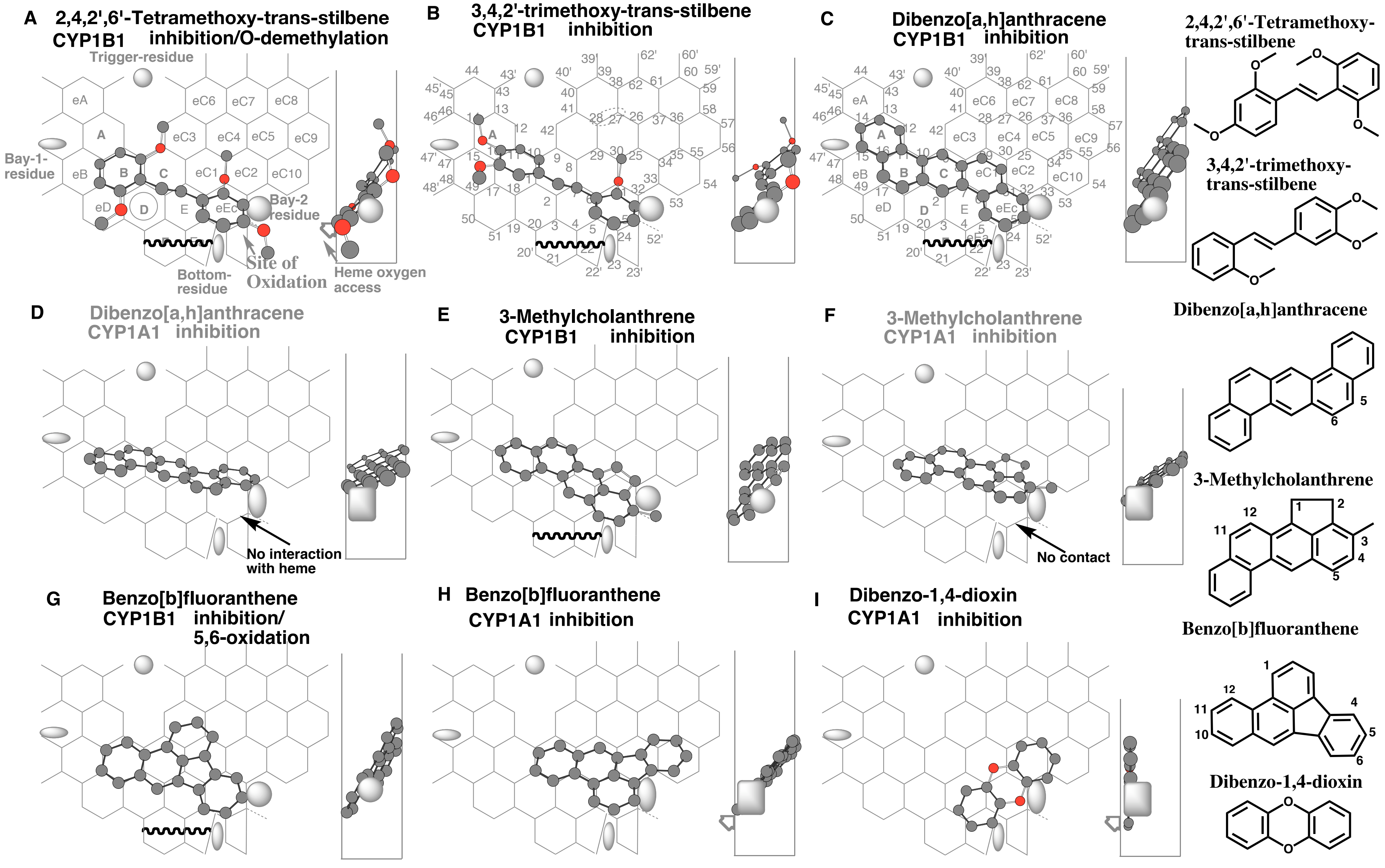

Supplement Figure 1

Placements of CYP1A1 and CYP1B1 inhibition

Placements on CYP1B1-Template of 2,4,2',6'-tetramethoxy-*trans*-stilbene for the inhibition/*O*-demethylation (A), of 3,4,2'-trimethoxy-*trans*-stilbene for the inhibition (B), of dibenzo[*a,h*]anthracene for the inhibition (C), of 3-methylcholanthrene for the inhibition (E), and of benzo[*b*]fluoranthene for the inhibition/5,6-oxidation are shown as cylindrical-shapes of 3D-structures on Template. Placements on CYP1A1-Template of dibenzo[*a,h*]anthracene (D), of 3-methylcholanthrene for the inhibition (F), of benzo[*b*]fluoranthene for the inhibition/11,12-oxidation (H) and dibenzo-1,4-dioxin inhibition (I) are also shown as cylindrical shapes of 3D-structures on Template. Functional and non-functional placements are indicated with dark- and grey-colored structure names, respectively. Bay-2 residues of CYP1A1 and CYP1B1 are shown as a grey oval and square in Width-gauge, respectively. 2D-structures are also shown with parts of chemical position numbers. Functional and non-functional placements are indicated with dark- and grey-colored structure names, respectively.
