# Supplementary material for: CYP1B1- and CYP1A1-Template systems and their application to metabolism and inhibition
Source: Genes Environ. 2025 Dec 26;48:1. doi: 10.1186/s41021-025-00351-x (PMC12781794; doi:10.1186/s41021-025-00351-x)
Supplement: Supplementary file 2 — Supplementary Material 2 [file 41021_2025_351_MOESM2_ESM.pdf]

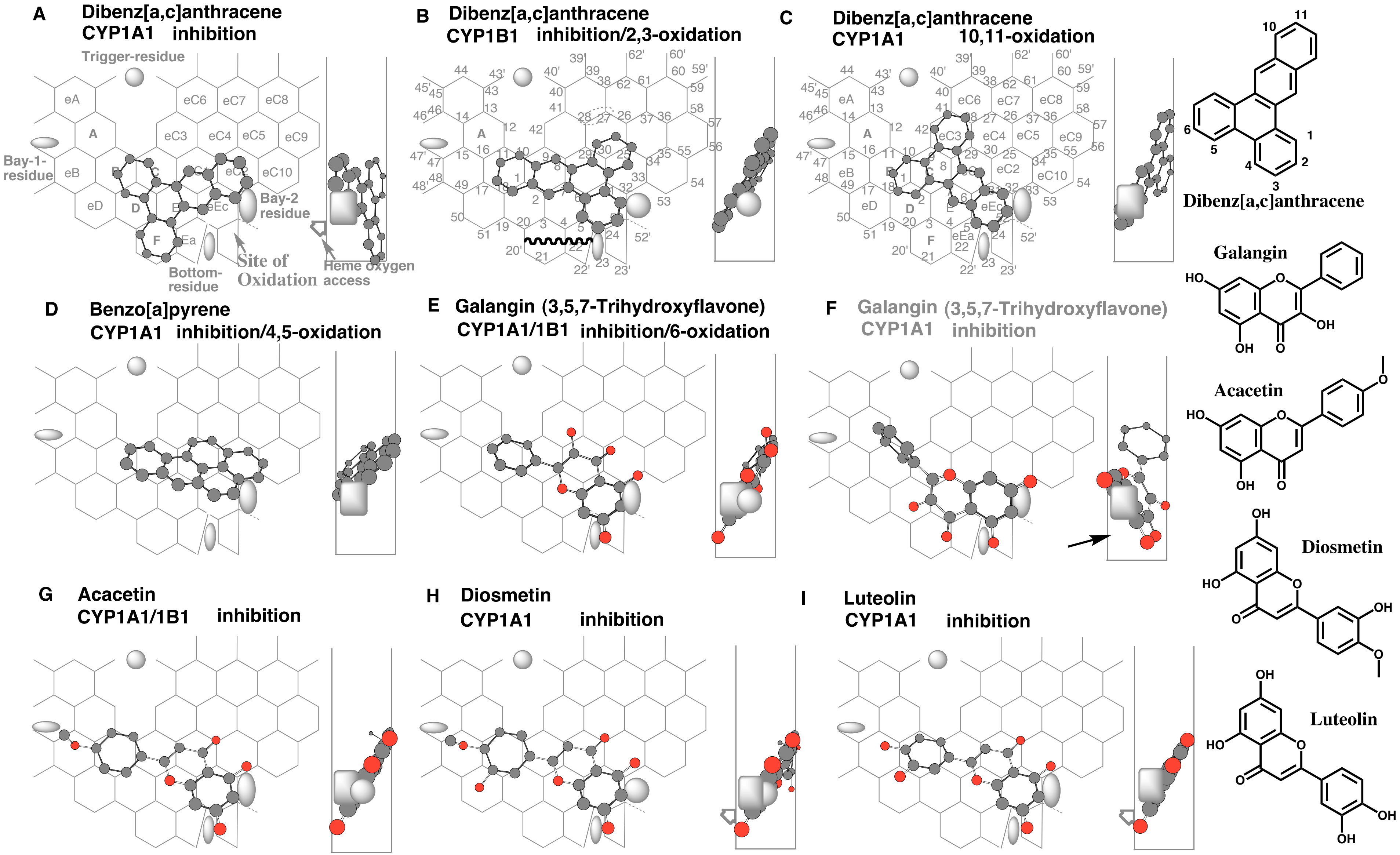

Supplement Figure 2

Placements of CYP1A1 and CYP1B1 inhibition 2

Placements of dibenz[*a,c*]anthracene for the inhibition/2,3-oxidation on CYP1A1-Template (A), and on CYP1B1-Template (B), for the 10,11-oxidation on CYP1A1-Template (C), of benzo[a]pyrene for the inhibition/4,5-oxidation (D), of galangin for the inhibition/6-oxidation (E and F), of acacetin for the inhibition (G), of diosmetin for the inhibition, and of luteolin for the inhibition (I) are shown as cylindrical-shapes of 3D-structures on Template. Functional and non-functional placements are indicated with dark- and grey-colored structure names, respectively. Bay-2 residues of CYP1A1 and CYP1B1 are shown as a grey oval and square in Width-gauge, respectively. 2D-structures are also shown with parts of chemical position numbers. Functional and non-functional placements are indicated with dark- and grey-colored structure names, respectively.
